# Exploring Knowledge and Sources of Information on Augmentative and Alternative Communication Among Board-Certified Behavior Analysts

**DOI:** 10.3390/bs16081402

**Published:** 2026-08-15

**Authors:** Alyssa Roylance, Sophia R. D’Agostino, Avery Briggs, Naima Bhana-Lopez

**Affiliations:** Department of Special Education and Rehabilitation Counseling, Utah State University, 2865 Old Main Hill, Logan, UT 84322, USA; sophia.dagostino@usu.edu (S.R.D.); avery.briggs@usu.edu (A.B.); naima.bhana@usu.edu (N.B.-L.)

**Keywords:** augmentative and alternative communication, board-certified behavior analysts, training, collaboration, mixed methods

## Abstract

Applied Behavior Analysis (ABA) is often recommended to help autistic individuals develop needed skills, and as such, Board-Certified Behavior Analysts (BCBAs) should be prepared to assist minimally verbal individuals to learn how to communicate. Augmentative and Alternative Communication (AAC) can be a helpful tool to assist communication for these individuals. An explanatory sequential mixed-methods study, including a survey with Likert-type and open-answer questions and a semi-structured interview, was conducted to learn if BCBAs are receiving training on AAC systems and where they are obtaining AAC-related professional development. The survey and interview also inquired about collaboration with other professionals regarding the use of AAC systems. The survey was analyzed using descriptive statistics, and the interview transcripts and open-response questions were analyzed using reflexive thematic analysis. The analysis of the survey answers, open-ended responses, and thematic analysis of the interviews indicated a familiarity with PECS, but participants differed in their knowledge of high-tech AAC. It was also indicated that BCBAs generally wanted more AAC training with a behavioral focus. Future research is needed to understand behavior analysts’ training needs and create effective training.

## 1. Introduction

Applied Behavior Analysis (ABA) therapy is often recommended to help autistic individuals develop a variety of skills, including functional, social, and communication skills (Gitimoghaddam et al., 2022; Rodgers et al., 2021). As such, Board-Certified Behavior Analysts (BCBAs) frequently work with autistic individuals who have communication deficits and may require assistive technology to communicate with others. It is estimated that 25% of autistic individuals will require communication supports for their entire life (Rose et al., 2016; Tager-Flusberg & Kasari, 2013). Augmentative and Alternative Communication (AAC) systems are used to help individuals communicate who may not be able to use vocal–verbal communication independently (Beukelman & Light, 2020). AAC includes aided and unaided tools. Unaided AAC does not necessitate any equipment (e.g., sign language, body language, gestures), while aided AAC requires supporting equipment (Beukelman & Light, 2020). Aided AAC includes the use of low-tech communication strategies (e.g., pictures, photographs, and the picture exchange communication system, or PECS), mid-tech (e.g., buttons with prerecorded messages), and high-tech communication strategies (e.g., applications on a tablet and dedicated speech-generating devices). Since AAC is frequently used for autistic individuals with communication needs (Mirenda & Iacono, 2009), it is recommended that BCBAs have experience and training on the use of AACs (Spencer et al., 2020).

The BACB ethics code (BACB, 2020) requires behavior analysts to practice within their scope of competence and to receive training before implementing new strategies (code 1.05). In order to understand the contextual factors around AAC and interventions with AAC, behavior analysts need proper training and education regarding evidence-based practices regarding AAC (Slocum et al., 2014). The code of ethics for behavior analysts also emphasizes the value of collaboration with other professionals (code 2.10). Effective collaboration deepens professional knowledge and can contribute to improved student outcomes (Koenig & Gerenser, 2006). When working with individuals with communication deficits, behavior analysts should collaborate with other professionals, particularly speech–language pathologists (SLPs), to determine the appropriate course of action. As SLPs are more likely to receive training on AAC systems (Clarke & Williams, 2020), this collaboration becomes even more beneficial. However, other invested parties, such as teachers and parents, also have helpful insights into how an AAC system is working for specific individuals (Chung & Stoner, 2016; Uthoff et al., 2021). These types of collaborators should be sought out when working with individuals who use AAC. Despite the need and benefits of collaboration, it is unclear if BCBAs are receiving training regarding AAC either during their higher education experience or during professional development after certification.

### Prior Research

There are several studies in the current literature base that demonstrate the evidence for using behavioral principles to teach the use of AAC. Lorah et al. (2024) conducted a systematic review of evidence-based practices used in AAC intervention research with autistic individuals. They found 38 studies that met their inclusion criteria. Several of the strategies they identified were rooted in ABA, including strategies such as prompting, differential reinforcement, discrete trial training, and naturalistic interventions. Findings indicated that practitioners who use AAC systems with autistic individuals use treatment packages instead of individual strategies, as most interventions researched used three or more evidence-based strategies within the intervention package. They also reaffirmed current research showing that instructional strategies are essential when implementing AAC systems and that the introduction of an AAC system is not enough alone.

The current literature base has a distinct lack of information on how therapists are trained to use AAC. For example, in a previous study, Clarke and Williams (2020) conducted a semi-structured interview similar to this current study regarding how speech–language pathologists (SLPs) received their training on AAC and how that affected the way they implement AAC with autistic children. Participants in this study primarily received training and education on the job and had limited education in autism or AAC during their preservice training. Barman et al. (2023) did a survey of 726 SLP graduate students and how prepared they felt to implement and assess AAC systems. Approximately one-third (n = 259) of these students had not taken a course on AAC systems, and approximately 40% of respondents (n = 309) indicated they did not feel prepared to work with AAC clients after graduation. Additionally, there are several articles discussing the training of paraeducators and special education teachers on specific interventions for the use of AAC systems (Andzik et al., 2021; Douglas et al., 2024; McCoy & McNaughton, 2021). However, to date, no study has investigated the pre-service training and in-service needs that behavior analysts receive and require on AAC.

It is essential to explore what BCBAs know about AAC systems and how they have learned about AAC in order to better support their understanding and use. As BCBAs deepen their understanding of AAC systems, they can more effectively support individuals with higher communication needs and collaborate more successfully with other professionals involved in AAC interventions. Therefore, the purpose of this pilot study was to explore BCBAs’ current training and understanding regarding AAC devices and how they received that training. Our research questions included the following. (1) What do BCBAs know about AAC systems? (2) Where did BCBAs receive their knowledge and understanding about AAC systems? (3) What topics did behavior analysts wish they knew more about in regard to AAC systems? (4) What effect does collaboration with other professionals have on BCBAs’ understanding of AAC systems?

## 2. Materials and Methods

### 2.1. Research Design

This study utilized an explanatory sequential mixed-method design (Creswell & Plano Clark, 2018). The initial quantitative phase included a survey, which was followed by a qualitative semi-structured interview phase. The interview results were used to build upon the results found from the survey.

### 2.2. Participants and Setting

Participants included 30 BCBAs and BCBA-Ds in the United States identified through purposive sampling. A purposive sampling strategy was used to recruit respondents who met the study’s inclusion criteria, allowing the survey to target individuals with relevant expertise and experiences rather than a representative sample of the general population (Palinkas et al., 2015). Those who completed at least 90% of the survey questions received a $10 Amazon e-gift card. Respondents who did not complete at least 30% of questionnaire items were excluded from the analysis. At the end of the survey, participants were able to indicate interest in participating in a semi-structured virtual interview. Interested individuals were emailed to set up a time to conduct the interview. Five participants completed the semi-structured interview.

### 2.3. Materials

The survey (see Supplementary Materials) was hosted on Qualtrics (Qualtrics, 2024, Provo, UT) and included 36 multiple-choice questions and 8 open-ended questions. Multiple-choice questions were formatted using a Likert-style scale. The survey included questions on the participants’ demographics, their comfort level with the use and training of others to use AAC, collaboration around AAC, perceived skill and understanding of AAC topics, and sources of their training and understanding regarding AAC. The survey also split questions regarding AAC training by master’s degree and doctoral degree when applicable. These questions were adapted from previous research on AAC and/or BCBAs’ training and education (Beaulieu et al., 2019; Da Fonte et al., 2022; Wormnæs & Abdel Malek, 2004). The survey questions were sent to experts in AAC to review and provide feedback on before recruiting participants.

The interviews were conducted on Zoom (Version 6.1.11) and were recorded with the aim of subsequently creating transcripts of the interviews. Participants were given seven topics to discuss regarding their experience learning about AAC.

1. Tell me about your experience with AAC that you received during your education.
2. Tell me about your experience with AAC that you received through professional development.
3. Tell me about your experience with implementing AAC.
4. Tell me about your experience with collaborating with other professionals about AAC.
5. Tell me about the barriers to implementing AAC.
6. Tell me about the facilitators (or aspects that help) for implementing AAC.
7. What do you recommend for other BCBAs that want to learn more about AAC?

The fourth topic was a potential follow-up topic, which was discussed if the participants had not discussed collaboration during their discussion implementing AAC. These topics were chosen to coordinate with the survey questions and were reviewed after receiving the survey answers. The fourth interview topic was added after reviewing the survey responses, but all other topics remained the same. Participants were emailed the questions ahead of time to allow them to consider the answers to their questions before the interview.

### 2.4. Procedure

This study was approved by an institutional review board, and all participants were required to sign informed consent forms before accessing the survey and before scheduling the interview. All survey data were de-identified prior to data analysis, and interviews were labeled by the date and time the interview took place instead of the participant’s name to increase confidentiality. To distribute the survey, emails with an overview of the study and a link to the survey were sent to third parties (ABA special interest groups and ABA companies) to share the research opportunity with BCBAs. Participants were given 1 month to complete the survey. The survey could only be taken once and took approximately 15 min to complete. The survey and interviews focused on the BCBAs’ self-perceived skills, comfort, and understanding regarding different aspects of AAC, as well as asking about how they received their understanding. As collaboration with other professionals can be an effective way to learn about AAC systems, several questions were also asked regarding collaboration. About 2 weeks after the first email, a reminder email was sent to the third parties to request they share the research opportunity again. Incentives were sent to qualifying participants within 2 weeks of completing the survey.

After the survey was closed, participants who indicated an interest in completing an interview were contacted. Those who indicated interest in the interview were also sent a reminder email after 2 weeks if they had not responded to the previous email. Interviews lasted from 16 min and 37 s to 29 min and 9 s, with an average of 23 min, and were held over a secure Zoom link. Zoom created an automated transcript from the video recording, which was reviewed by a researcher for accuracy and de-identified before analysis. Interview transcripts were sent back to the individual interviewed as a member check.

### 2.5. Data Analysis

Descriptive statistics were used to analyze answers to Likert-style questions from the survey. Frequency tables were created for these questions using IBM SPSS Statistics (Version 29). The first and third authors used reflexive thematic analysis as described by Braun and Clarke (2006) to identify themes across participant interviews. These authors started by documenting their positionality to acknowledge any bias that could come from their personal experiences. Then, they began by reading over the transcripts to familiarize themselves with the interviews and noted initial ideas. These ideas were then discussed, and codes were created. The transcripts were re-read with the codes in mind, and data from the interviews was gathered to support each of the codes. These codes were then discussed and collated into potential themes. Following initial coding, the research team generated 19 candidate themes that captured distinct patterns within the data. These candidate themes were then reviewed iteratively to evaluate their internal coherence, conceptual distinctiveness, and relevance to the research questions, consistent with recommendations for reflexive thematic analysis (Braun & Clarke, 2006). During this phase, themes with overlapping meaning were collapsed or reorganized into broader thematic areas representing Sources of Training, Facilitators, and Barriers. Decisions regarding theme boundaries and organization were made through iterative discussions among the research team, with reference to the coded data and the complete interview transcripts, until agreement was reached that the thematic structure accurately represented participants’ experiences. Finally, the themes were organized into a thematic map to illustrate potential relationships between themes and subthemes. The second author examined the completed thematic map and interviews as an external validity check, and two more themes were added to the map as a result. Participants who completed the survey were also provided with open-response opportunities to provide more information about each section of questions. The themes were originally created from the interview responses, and then the open responses were checked for further themes. No new themes were found from the open responses in the survey.

### 2.6. Researcher Positionality

When conducting qualitative analyses, we recognize that biases can affect the conclusions and connections made, and as such, we wish to be transparent about the backgrounds of the authors of this paper. The first author is a BCBA, while the second and fourth authors are BCBA-Ds. Three authors are also special education teachers. Two authors identify as neurodiverse, and all authors have worked with children who have used AAC systems in their professional line of work.

## 3. Results

### 3.1. Quantitative Data Results

This section provides the results from the quantitative survey questions, including demographics of the participants and Likert scale responses about the participants’ perceptions of importance, skill, training, collaboration, and understanding around AAC systems.

#### 3.1.1. Participant Demographics

Participant demographics are displayed in Table 1. The majority of participants identified as white and female, which aligns with current BCBA demographics reported by the BACB (2025). The most common role reported was supervisor; most respondents had worked between 1 and 5 years as a BCBA, and a majority of the respondents indicated they worked with autistic individuals.

#### 3.1.2. Importance, Skill, and Training

Table 2 depicts participants’ responses to the Likert scale survey questions. Participants were split regarding comfort and skill teaching others how to use AAC, with just over half indicating they felt “moderately comfortable or skilled” or “very comfortable or skilled” (n = 16) and the rest indicating they were only fairly comfortable, uncomfortable, or “very uncomfortable or skilled” (n = 14). A grand majority of participants (n = 26) agreed that it was very important to receive training and education on AAC. Participants also indicated a lack of education and training around AAC during their formal education experiences, with 23 indicating little to no material encountered. When asked about professional training after certification, most participants received little to no CEU credits (n = 19), had reduced opportunities to receive CEU credits (n = 23), and had little to no training from their employers (n = 22). Over half of the participants (n = 17) indicated they received their knowledge and skills by working with individuals who use AAC frequently or throughout their career.

#### 3.1.3. Ease and Comfort of Collaboration

Participants (n = 24) indicated they were at least moderately comfortable collaborating with others regarding AAC. However, they were split regarding ease of finding someone to collaborate with and similarly split regarding gaining knowledge through collaboration with other professionals, with a little over half (n = 16) indicating frequent collaboration and the rest (n = 13) indicating minimal collaboration with other professionals.

#### 3.1.4. Understanding of AAC Concepts

When the respondents were asked about specific concepts regarding AAC, 16 participants indicated little to no understanding of AAC assessment, and 14 participants indicated little to some understanding of vocabulary selection and types of symbols. The responses were split regarding AAC instruction, with 14 indicating little to some understanding and 16 indicating moderate to extensive understanding. Most participants (n = 20) indicated moderate to extensive knowledge of low-tech AAC systems, and many (n = 18) also indicated high levels of knowledge in high-tech AAC systems.

### 3.2. Themes from Qualitative Data

This section provides the results of the qualitative data collected during the interview process. Examples of quotes corresponding with the themes can be found in Table 3.

#### 3.2.1. Sources of Training

**Figure 1:**
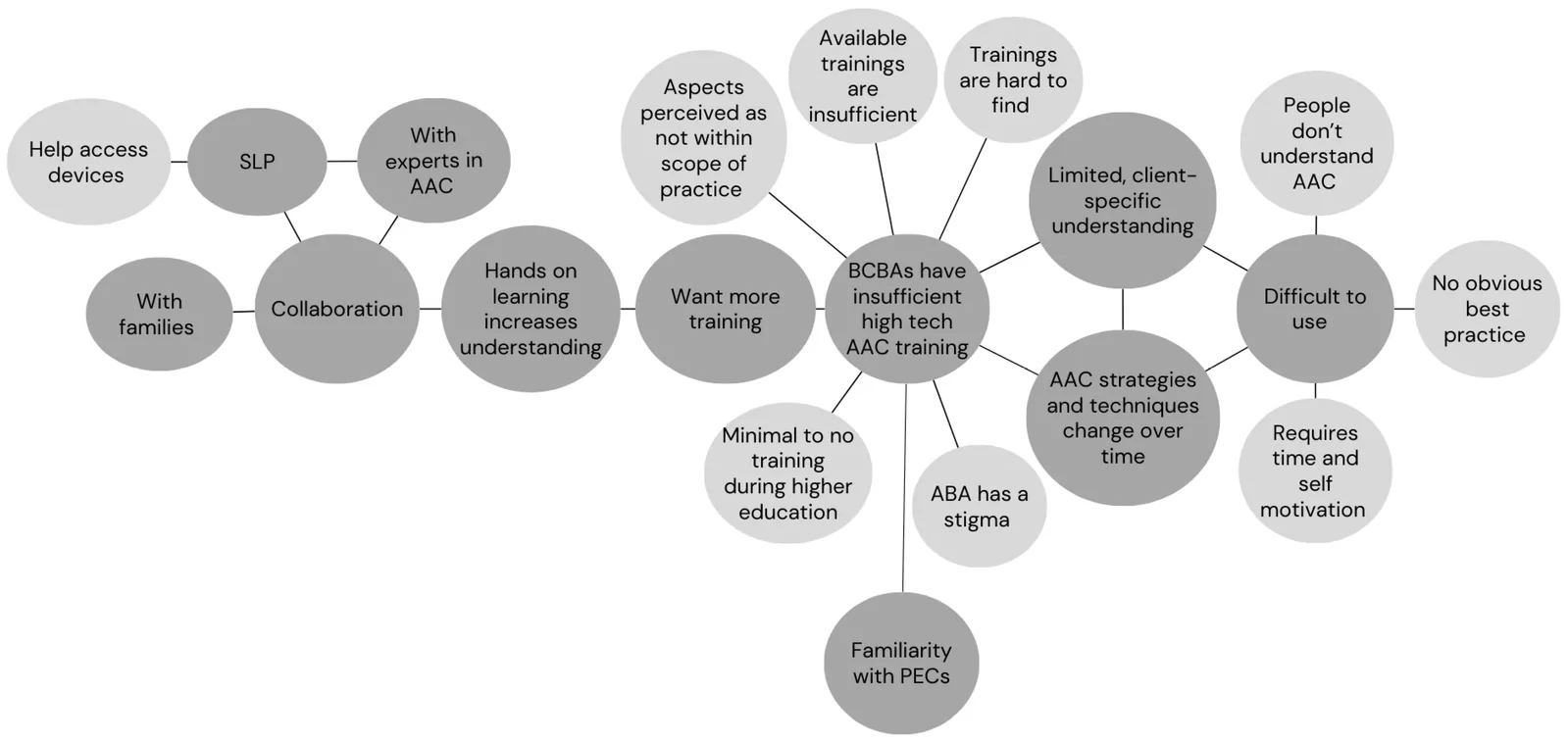
Thematic map created from the themes gathered during this study using Canva (https://www.canva.com/, accessed on 18 October 2024).

Themes from analysis of the interviews can be found in the thematic map in Figure 1. Participants frequently indicated familiarity and comfort with low-tech AAC options such as PECS. The limited training opportunities on AAC that were available were often limited to PECS. One interviewee stated the following:

I guess the fact that PECS is so clearly laid out, the step-by-step process makes it not intimidating to try to implement… So it makes it a little easier for you to feel confident like I can do this, and I know what I know what I’m doing enough to know that whatever I’m going to do is going to help the child and not hurt them in any way.

However, when it came to high-tech AAC options, the reverse appeared to be true. BCBAs indicated a current lack of training regarding high-tech AAC and a desire for more training to be available, as well as more in-depth training. One participant said as follows:

We have more and more kids coming in with an AAC device like that’s been taught since they were like 18 months through their regional center program. And so I would be the barrier to their trajectory of growth if I don’t know how to utilize it.

Participants mentioned receiving little to no training on high-tech AAC during their coursework to become a BCBA, with only a couple having had a class or part of a class discussing high-tech AAC. One participant chose to complete a project about PECS, but the original assignment was open-ended and did not require the participant to look at AAC. This demonstrates the need for self-motivation in order to learn about AAC as a BCBA.

Participants also indicated that when they searched for high-tech AAC trainings for BCBAs in the past, they struggled to find any. They also found the available trainings to be lacking in the knowledge they desired regarding teaching AAC behaviorally: “A lot of the AAC CEUs that I’ve seen and have done, I’ve only done one or 2, but they weren’t detailed enough or specific enough for me to apply those skills in my setting.” It was unclear to participants if these trainings did not exist or if they were simply difficult to find. “There’s just not a lot of, I think, instruction or continuing education focus strictly on AAC device usage correctly from like BCBAs.”

#### 3.2.2. Facilitators

**Hands-on learning.** When asked about facilitators to underuse and using high-tech AAC, participants’ main suggestion was to collaborate with experts in AAC. They discussed several personal experiences working with SLPs or parents to learn about their clients’ devices and often mentioned that was where they learned the most about high-tech AAC use. They found that hands-on learning experiences led to the best understanding, especially when an expert, typically an SLP, was able to watch them implement the devices and give feedback. “I think definitely, seeing it in action has been helpful. And then having again the SLP shadow me, or someone who’s more well versed in it shadow me to see if I’m doing it correctly.”

**Collaboration.** Participants also discussed the importance of collaborating with SLPs due to their expertise and their ability to complete assessments and get funding for the devices, which BCBAs are not currently able to do. “And an SLP to test for, that company that we used like SLP has to do like a whole assessment of which app is the best, and things like that to make sure it’s individualized to that specific client.” Participants noted that if a company had a BCBA or other professional who was an expert in AAC, then that would be beneficial to increase understanding and accessibility: “And then hopefully, if you have someone in your immediate community like a BCBA who is familiar with the AAC device just to like, collaborate and learn from them, too.”

#### 3.2.3. Barriers

**ABA Has a Stigma.** A few participants mentioned accessing trainings for SLPs regarding high-tech AAC to increase their understanding. However, it was also stated that occasionally the other professionals in the training would be hesitant to collaborate due to the stigma ABA can have:

When I did the SLP CEU as a BCBA, there was a lot of, you know, negativity around that as well. So I think there wasn’t as much collaboration as I’d hoped for, because there was already this pretense of like, nope, like you’re doing the wrong thing, we’re here to do the right thing.

The negative opinion of ABA as a field was discussed as a barrier to AAC collaboration in other situations as well when attempting to collaborate with parents or other professionals to learn more about an individual’s specific AAC use.

I do coordinate with outside SLPs and outside OTs. For my clients here, because we work in a group setting and when they’re not like in house SLPs and OTs, there is a bit of a hesitation already when I call them to get those collaboration and coordination of care. I think once they realize we are more of a preschool, and there’s a little bit more like early childhood education as our background before we even implement the science of ABA. Then—I can physically hear their voice relaxing and being willing to and wanting to collaborate with me.

**Scope of Practice.** There was also a question of which aspects of AAC use were within the scope of practice of BCBAs. Some participants mentioned they would not be comfortable choosing a high-tech AAC device independently and would require an SLP or other expert to do so because that does not fall within their scope of practice. This concern was also brought up regarding starting a high-tech AAC program in general, but that they felt they could help once the program had been started. One participant stated, “I feel like it’s kind of out of my scope at the moment to really suggest [starting an AAC].”

**Limited, Client-Specific Understanding.** Some of the barriers discovered from the qualitative data include that high-tech AAC strategies and techniques change over time, and often BCBAs only have limited, client-specific understanding of high-tech AAC. Some BCBAs were taught to use high-tech AAC in one way but were taught more evidence-based techniques later in their career. As an example, participants mentioned being trained to use AAC as more of a prompting procedure initially, but then several years later were taught to consider the device as the individual’s voice and that it should not be taken away. Participants also discussed learning a client’s specific program and how it was used but not feeling confident in their ability to start that same program with a new client: “And so with that client, I learned that client specific use of the AAC device… I don’t know if I have the confidence to like use AAC device for any child.”

**Difficult to Use.** High-tech AAC systems were also discussed as being difficult to use. Participants who wanted to understand AAC often had to use their own time and resources to learn more about each program their client was using. This was partially due to a lack of company-provided training but also due to different programs having different controls and setups that required more effort to understand. One participant said, “So to be able to use that fluidly in our session is something I’m taking upon myself to learn now, just for this client.” The participants also felt that there was not an obvious best practice for implementing high-tech AAC: “I don’t see one like best practice from any of those fields. I mean our field, either.” Another participant mentioned they wished there were stages to follow similar to PECS, as that would make progressing to more complex language with high-tech AAC easier. One comment from the open-response questions on the survey also pointed out that sometimes the other professionals they tried to collaborate with were also not trained in AAC or were not familiar with autism, which made the collaboration less beneficial.

**Lack of Understanding by Others.** These BCBAs also recognized that there is a general lack of understanding of high-tech AAC by people in the user’s life, which can make it difficult for these skills to generalize. When asked about potential barriers to AAC use, one participant stated, “Also, I think probably other people’s understanding of what and how to use them.” This included people such as families, other individuals AAC users may interact with outside of therapies, and other BCBAs. It is not always easy to know how to be an effective communication partner with someone who has a unique way of communicating, which can reduce effective practice and the number of opportunities for AAC users to communicate. Each of these variables contributes to high-tech AAC systems being considered difficult to use.

### 3.3. Integration

Overall, the qualitative findings largely converged with the survey results and provided additional context regarding why participants perceived gaps in their preparation to implement AAC. Survey respondents reported limited formal instruction during graduate education, few continuing education opportunities, and minimal employer-provided training, despite rating AAC training as highly important. The interviews expanded on these findings by illustrating that available training was often limited to low-tech AAC systems, particularly PECS, while instruction on high-tech AAC was described as difficult to locate, insufficiently detailed, or not designed for behavior analysts.

The qualitative findings also helped explain patterns observed in the survey regarding collaboration. Although survey respondents generally reported feeling comfortable collaborating with other professionals, they were divided regarding how easily collaboration could be established and the extent to which collaboration contributed to their AAC knowledge. Interviews demonstrated that collaboration, particularly with speech–language pathologists, served as one of the most valuable sources of learning through hands-on practice, modeling, and performance feedback. At the same time, participants described barriers that could limit these collaborative relationships, including differing perceptions of professional roles, uncertainty regarding scope of practice, and stigma associated with ABA. These qualitative findings clarify why collaboration was viewed as both highly valuable and, at times, difficult to access.

Finally, the interviews extended the survey findings by identifying barriers that were not directly measured quantitatively. Participants described high-tech AAC systems as complex, rapidly evolving, and often requiring client-specific knowledge that did not readily generalize to new learners. They also emphasized the need for ongoing self-directed learning, concerns regarding determining appropriate AAC systems within their scope of practice, and limited understanding of AAC among families and other communication partners.

## 4. Discussion

This study aimed to explore the experiences of BCBAs providing AAC supports. We asked specific survey questions related to BCBAs’ training experience during preservice education and in-service training (e.g., CEUs or conferences), BCBAs’ confidence implementing AAC components, and their perceptions of collaboration with other professionals involved in AAC systems. We also conducted follow-up interviews with a subset of participants to further explore their experiences implementing AAC systems. As BCBAs typically work with autistic individuals who need communication supports, understanding what BCBAs know about AAC systems can highlight areas where more support can be provided to BCBAs regarding AAC systems. These findings highlighted the desire for more AAC training, the need to make these trainings easily accessible, and the benefits of collaborating with other professionals.

### 4.1. BCBAs’ Knowledge of AAC Systems

Survey results indicated that BCBAs view understanding AAC systems as an important skill; however, many reported limited confidence in independently implementing these systems. Participants’ comfort with PECS suggests that prior experience functioned as a significant facilitator of BCBAs’ perceived understanding and confidence in AAC. Additionally, most survey participants reported high confidence in their ability to implement and teach others how to use low-tech AAC systems, such as PECS. This is much higher than the reported confidence in implementing and teaching others about high-tech AAC. Behavior analysts frequently need to teach others, such as behavior technicians and family members, how to use communication strategies (Fisher et al., 2020), and this gap in confidence to teach should be addressed. These findings are supported in the literature, as behavior analysts in previous studies consistently identified PECS as a familiar and well-established evidence-based practice (Bondy & Frost, 1994; Marshall et al., 2023; Pruneti et al., 2024). Based on the interviews, high-tech AAC trainings were reported to be difficult to find and access, whereas PECS-related trainings are easily found on the PECS website, increasing their accessibility (Pyramid Educational Consultants, n.d.). During interviews, participants emphasized that PECS was “easy” to use because of its clearly defined, step-by-step instructional model, which aligns closely with behavioral principles and instructional practices commonly used by BCBAs such as task analyses (Cooper et al., 2020; McConomy et al., 2022). Importantly, several participants described generalizing this structured PECS framework when teaching high-tech AAC systems, which has also been shown to be effective in the literature (Gilroy et al., 2017, Genc-Tosun et al., 2025), suggesting that familiarity with PECS may serve as a conceptual bridge to more complex AAC technologies. This overlap likely contributed to participants’ overall confidence in their AAC knowledge, even when formal training in high-tech AAC was limited. These findings suggest that leveraging BCBAs’ existing PECS knowledge may be a powerful entry point for AAC training, particularly in contexts where access to specialized AAC expertise is constrained (Koenig & Gerenser, 2006; LaFrance et al., 2019; Spencer et al., 2020).

### 4.2. Sources of Knowledge About AAC Systems

A major barrier cited by participants was the availability of high-tech AAC training for behavior analysts. Kranak et al. (2023) found that when BCBAs obtained continuing education units (CEUs), they most often used virtual conferences, webinars, or in-person conferences in close proximity, which indicates that convenience in accessing CEUs may influence which CEUs are chosen. Thus, if trainings or CEUs are difficult to find, behavior analysts are less likely to engage with those trainings. This may lead to a reliance on learning how to use high-tech AAC on the job instead of through structured training opportunities. However, as discussed in the interview process, those who had learned how to use high-tech AAC with one individual did not feel confident in generalizing that knowledge to other individuals. This was also demonstrated in a lack of confidence implementing AAC systems to teach more complex language beyond basic requesting. Participants commented on both the open-response questions in the survey and the interviews that they wished there was more guidance on how to use and progress with high-tech AAC, like the PECS model, despite the prevalence of AAC research demonstrating several strategies for teaching AAC instruction that align with ABA principles (Lorah et al., 2024).

Many BCBAs who took the survey indicated they felt comfortable teaching others how to use AAC, so more collaboration among behavior analysts is needed to support those who lack AAC expertise. More centers could support training one behavior analyst in these known strategies and techniques so they can return and train the other analysts, like in a pyramidal training model (Maffei-Almodovar et al., 2025).

### 4.3. Current Limitations of AAC Knowledge and Training

Some participants expressed ethical and professional concerns about using high-tech AAC, such as uncertainty about whether assessment and progression fell within their scope of practice. These concerns were often tied to perceived limits in the scope of competence, which is defined as “the professional activities a behavior analyst can consistently perform with proficiency” by the BACB ethics code (BACB, 2020, p. 8). This was reflected in this study’s findings, as participants cited insufficient training or expertise rather than viewing AAC use as outside their professional role. A major facilitator for comfort and understanding AAC systems was access to SLPs and AAC experts, which becomes even more important when BCBAs have not received the training for AAC systems to be within their scope of competence.

Many participants indicated their desire for more high-tech AAC training. This may be a result of the lack of preservice training on AAC systems, which requires BCBAs to spend their own time and resources learning how to use different AAC systems. Findings mirror those by Clarke and Williams (2020), who investigated SLP training on AAC systems and found that SLPs were also not being taught about AAC during their preservice training. Barman et al. (2023) found that SLPs who did not receive AAC training during their graduate program felt less confident in working with individuals who use AAC, but those who did receive preservice training felt more confident. It would be beneficial to include more opportunities in preservice courses for a variety of professionals to learn about AAC systems. This could be facilitated by including classes on AAC systems into programs teaching BCBA coursework as part of teaching valid alternative behaviors or collaboration (H.2, H.3, H.8; BACB, 2024) or by creating a micro-credential in AAC systems (Ahsan et al., 2023).

### 4.4. Collaboration

Collaboration with SLPs emerged as a key facilitator for the implementation of high-tech AAC systems. Eighty percent of survey respondents reported feeling comfortable collaborating with other professionals about AAC. Participants who reported access to SLPs or AAC specialists described greater confidence and competence in AAC use, attributing this to opportunities for real-time consultation, guided decision-making, and individualized feedback. Interview data indicated that hands-on experience with AAC devices, coupled with expert modeling and coaching from SLPs, reduced uncertainty and increased implementation fidelity. Given SLPs’ specialized training in communication development and AAC system selection, these findings underscore the value of interdisciplinary collaboration in supporting effective AAC implementation (Spencer et al., 2020). This facilitator highlights the importance of structural supports like intentional collaboration models or consultation frameworks, especially for rural BCBAs who may have limited access to AAC expertise. This aligns with our understanding of interprofessional collaboration between BCBAs and SLPs (Lane & Brown, 2023). Koenig and Gerenser (2006) discussed how collaboration with other professionals can increase productivity, as combined knowledge across their fields prevents time spent reinventing the wheel, such as processes to increase motivation through ABA principles or the evidence base for speech-generating devices from SLPs.

Close contact with SLPs may also lead to better training in AAC systems when those SLPs have expertise in AAC systems. The demand for individuals who are able to bridge this gap is high enough that in 2005 a Speech Pathology–Applied Behavior Analysis (SPABA) special interest group was created to help connect individuals who are interested in the collaboration of these two fields, according to this special interest group. Furthermore, as of May 2025, 490 active professionals were dually certified as behavior analysts (BCBA, BCaBA, or QBA) and SLPs (SPABA, 2025). Those professionals valued the expertise of both fields enough to take the time to receive the education and training needed to become dual-certified. Continued close collaboration between these fields can allow both professions to learn and grow from each other, especially in the context of AAC systems.

However, it was noted by several participants that if there was not an expert already in the company, getting access to those experts could be difficult. Participants discussed how SLPs often have a long waitlist, which has been found to be up to a 42-month wait (McGill et al., 2021), which can make collaborations even more difficult. Occasionally, when they did get access to an SLP, the SLP lacked expertise in either AAC systems or autism. This aligns with the findings of Barman et al. (2023) and Clarke and Williams (2020), which found that many SLPs are not receiving AAC training in their preservice training. Given our findings that BCBAs had also engaged in little to no formal training regarding AAC, especially high-tech AAC, we can conclude that many behavior analysts were likely to be practicing outside their scope of competence when their caseloads included individuals who used high-tech AAC (Slocum et al., 2014). As BCBAs are ethically required to practice within their competence (BACB, 2020), BCBAs should continue to search out training and other sources of developing expertise when the individuals they work with need extra support in communication.

### 4.5. Limitations and Future Research Directions

Limitations of this study include the low diversity of participants. Although the participants generally represent the majority of BCBA certificants (i.e., white and female), it is also important to assess the needs of the minorities so we can support their unique needs as well. This study also did not ask about participants’ state of residence, which could have affected the responses, as different states may have different preservice, licensure, or continuing education requirements, as well as differential access to experts in AAC. Future research should continue to assess BCBAs’ knowledge and use of AAC systems among a larger sample of behavior analysts across a variety of settings and locations. It would be particularly beneficial to assess BCBAs’ knowledge of AAC systems when they do not have easy access to an SLP. We also see some contradictory results between the quantitative results and the qualitative results about the comfortability of high-tech AAC, which should be further investigated. Additionally, some survey questions, such as comfort collaborating with or skills teaching AAC systems, did not distinguish between low-tech and high-tech AAC systems. A future study that separates these two types could yield more specific insights and allow for a more detailed discussion of results related to each. The familiarity with PECS within the ABA field may have increased ratings of comfort with AAC systems overall, so if high-tech AAC systems were rated separately, their ratings may have been lower. Finally, it may be beneficial to assess other non-PECS low-tech AAC systems separately to determine behavior analysts’ knowledge and comfort with those systems.

## 5. Conclusions

These findings show that there is room for improvement in BCBAs’ understanding of AAC systems, particularly with high-tech AAC. Most BCBAs are not trained during their higher education and have to find their own training. Many reported not knowing much about the specifics of AAC systems, such as assessment, vocabulary selection, and the use of symbols. However, collaboration with other professionals and increased hands-on experience can be a major facilitator. The population behavior analysts work with frequently need communication support or already use AAC systems, which makes it an important skill for behavior analysts to acquire. As a result, many expressed a desire for additional behavior analytic training focused on high-tech AAC to better support individuals with complex communication needs. Behavior analysts who do not have knowledge of AAC systems can unintentionally become the barrier to success for individuals with communication needs. This also highlights the importance of effective collaboration among professionals to support individuals with complex communication needs. Future research should continue to assess how to help BCBAs who do not have an understanding of AAC systems and potential barriers to receiving that understanding. Overall, these findings show the importance of effective and accessible training for AAC systems for behavior analysts as the number of people who use these systems to communicate increases.

## Figures and Tables

**Table 1:** Demographics.

Demographic Characteristic	%	N
Age		
18–29	40.0	12
30–39	46.7	14
40–49	10.0	3
50–59	3.3	1
Gender		
Male	6.7	2
Female	93.3	28
Race (select all that apply)		
White	86.7	26
Black/African American	3.3	1
Asian	13.3	4
Mixed	3.3	1
Ethnicity		
Hispanic, Latino, or Spanish origin	16.7	5
Non-Hispanic, Latino, or Spanish origin	80.0	24
Primary role		
Practitioner (direct services)	23.3	7
Supervisor	53.3	16
Administrator	16.7	5
Professor/researcher	3.3	1
* Combination of above	3.3	1
Years as a BCBA		
<1	10.0	3
1–5	53.3	16
5–10	30.0	9
10–15	6.7	2
Primary population worked with		
Autism Spectrum Disorder	86.7	26
Special Education	6.7	2
Mental Health	3.3	1
* Other (unspecified)	3.3	1
Primary work setting		
Public school	6.7	2
Private school	6.7	2
Client’s home	3.3	1
Center or clinic	70.0	21
Hospital community	3.3	1
* Clinic and university	3.3	1
* Home and clinic	3.3	1
* Nonprofit	3.3	1
Field of master’s degree		
Applied Behavior Analysis	60.0	18
Behavior Analysis	6.7	2
Education	23.3	7
School psychology	3.3	1
* Special Ed w/BA emphasis	3.3	1
* Special physical education	3.3	1
Field of PhD		
Applied Behavior Analysis	3.3	1
N/A—no PhD	96.7	29

* Indicates the participant typed in the specified response.

**Table 2:** Responses to survey questions.

Questions and Answers with at Least One Response	%	N
How many total years of experience do you have providing AAC to at least one individual (includes work as an RBT, teacher, or other relevant professional experience)?		
<1	6.7	2
1–5	56.7	17
5–10	23.3	7
10–15	13.3	4
How comfortable do you feel teaching someone how to use augmentative and alternative communication strategies (AAC)?		
Very uncomfortable	6.7	2
Uncomfortable	10.0	3
Fairly comfortable	30.0	9
Moderately comfortable	33.3	10
Very comfortable	20.0	6
How comfortable do you feel collaborating with someone to determine how to use AAC?		
Very uncomfortable	3.3	1
Uncomfortable	6.7	2
Fairly comfortable	10.0	3
Moderately comfortable	30.0	9
Very comfortable	50.0	15
How easy or difficult do you feel it is to find someone to collaborate with on the use of AAC?		
Very difficult	6.7	2
Difficult	16.7	5
Fairly comfortable	36.7	11
Moderately comfortable	10.0	3
Very comfortable	30.0	9
How skilled do you feel teaching someone how to use AAC?		
Unskilled	16.7	5
Fairly skilled	26.7	8
Moderately skilled	40.0	12
Very skilled	16.7	5
How important do you feel it is to receive training and education on how to use AAC?		
Neutral	3.3	1
Moderately important	10.0	3
Very important	86.7	26
To what extent do you have knowledge and skills in AAC assessment?		
None	13.3	4
Little (I have heard of it, but have not implemented it)	40.0	12
Some (I have some knowledge and implemented it a few times)	16.7	5
Moderate (I understand it and implement it regularly)	20.0	6
Extensive (I am proficient in knowledge and skills and could teach someone else)	10.0	3
To what extent do you have knowledge and skills in AAC instruction?		
Little (I have heard of it, but have not implemented it)	16.7	5
Some (I have some knowledge and implemented it a few times)	30.0	9
Moderate (I understand it and implement it regularly)	36.7	11
Extensive (I am proficient in knowledge and skills and could teach someone else)	16.7	5
To what extent do you have knowledge and skills in vocabulary selection?		
Little (I have heard of it, but have not implemented it)	31.0	9
Some (I have some knowledge and implemented it a few times)	31.0	9
Moderate (I understand it and implement it regularly)	27.6	8
Extensive (I am proficient in knowledge and skills and could teach someone else)	10.3	3
To what extent do you have knowledge and skills in types of symbols?		
None	10.3	3
Little (I have heard of it, but have not implemented it)	31.0	9
Some (I have some knowledge and implemented it a few times)	31.0	9
Moderate (I understand it and implement it regularly)	24.1	7
Extensive (I am proficient in knowledge and skills and could teach someone else)	3.4	1
To what extent do you have knowledge and skills in no/low tech AAC systems (e.g., PECS)?		
None	3.4	1
Little (I have heard of it, but have not implemented it)	6.9	2
Some (I have some knowledge and implemented it a few times)	20.7	6
Moderate (I understand it and implement it regularly)	27.6	8
Extensive (I am proficient in knowledge and skills and could teach someone else)	41.4	12
To what extent do you have knowledge and skills in high tech AAC systems (e.g., speech generative devices, apps such as Proloquo2Go or TD Snap)?		
Little (I have heard of it, but have not implemented it)	13.8	4
Some (I have some knowledge and implemented it a few times)	24.1	7
Moderate (I understand it and implement it regularly)	44.8	13
Extensive (I am proficient in knowledge and skills and could teach someone else)	17.2	5
During your master’s degree education, how much material in your behavior analytic coursework was dedicated to teaching you to use AAC?		
None	40.0	12
Little (one article)	36.7	11
Some (part of a class period)	6.7	2
Extensive (several class periods or more)	13.3	4
Not sure	3.3	1
During your master’s degree education, how much material in your non-behavior analytic coursework was dedicated to teaching you how to use AAC?		
None	69.2	18
Little (one article)	19.2	5
Some (part of a class period)	3.8	1
Extensive (several class periods or more)	3.8	1
Not sure	3.8	1
During your doctoral degree education, how much material in your behavior analytic coursework was dedicated to teaching you how to use AAC?		
None	100	4
During your doctoral degree education, how much material in your non-behavior-analytic coursework was dedicated to teaching you how to use AAC?		
None	100	3
During your master’s degree education, how much hands-on training (e.g., fieldwork, practicum) was dedicated to teaching you how to use AAC?		
None	31.0	9
Little (one instance)	27.6	8
Some (one training)	13.8	4
Moderate (several trainings)	27.6	8
During your doctoral degree education, how much hands-on training (e.g., fieldwork, practicum) was dedicated to teaching you how to use AAC?		
None	100	3
Since becoming certified, how much continuing education (CE) have you received teaching you how to use AAC?		
None	34.5	10
Little (1–3 credits)	31.0	9
Some (3–5 credits)	17.2	5
Moderate (5–10 credits)	10.3	3
Extensive (11+ credits)	6.9	2
Since becoming certified, how many continuing education (CE) opportunities have you seen offered at behavior-analytic conferences or online teaching you how to use AAC?		
None	27.6	8
1–2 per year	51.7	15
3–4 per year	17.2	5
5–8 per year	3.4	1
How much training does your employer provide you on how to use AAC?		
None	41.4	12
Little (I was shown how to by a colleague a couple of times)	34.5	10
Some (I received one training session)	6.9	2
Moderate (I received several training sessions)	13.8	4
Extensive (I received several training sessions and extensive feedback while implementing AAC)	3.4	1
To what extent did you obtain your knowledge and skills through personal experience?		
None	3.4	1
Little (I worked with one individual who used AAC)	10.3	3
Some (I worked with a few individuals who use AAC)	27.6	8
Moderate (I have frequently worked with individuals who use AAC)	37.9	11
Extensive (I have worked with individuals who use AAC throughout my whole career)	20.7	6
To what extent did you obtain your knowledge and skills through collaboration with other professionals, such as speech language pathologists (SLPs) or teachers?		
None	3.4	1
Little (I had one interaction with a professional regarding AAC)	34.5	10
Some (I received a full training from another professional regarding AAC)	6.9	2
Moderate (I have collaborated with other professionals several times regarding AAC)	27.6	8
Extensive (I frequently collaborate with other professionals regarding AAC)	27.6	8
To what extent did you obtain your knowledge and skills through other sources (colleagues, TV presentations, journals, books)?		
None	6.9	2
Little (I read one article on AAC)	20.7	6
Some (I have read a couple articles or a book on AAC)	34.5	10
Moderate (I have read many articles or a couple books on AAC)	31.0	9
Extensive (I have read many articles and read several books on AAC)	6.9	2

**Table 3:** Definitions of major themes and quotes for subthemes.

Themes	Definition of Major Themes and Quotes for Subthemes
**Sources of Training**	Defined as the settings where trainings on AAC strategies and techniques are being taught or could be taught, the process of finding those trainings, or the effectiveness of those trainings. These trainings can include AAC strategies and techniques such as understanding high tech vs. low tech systems or techniques to increase the use of an AAC.
Familiarity with PECS	And I really approach all AAC similarly to PECs now, like even more high tech options with iPad devices and things like that. (Respondent 3, 25 June 2024, 12 p.m.) I guess the fact that PECS is so clearly laid out, I guess the step-by-step process makes it not intimidating to try to implement… So it makes it a little easier for you to feel confident like I can do this, and I know what I know what I’m doing enough to know that whatever I’m going to do is going to help the child and not hurt them in any way. (Respondent 1, 24 June 2024, 12:00 p.m.)
BCBAs have insufficient high tech AAC training	I wish I had more training on how to use an AAC effectively with my clients. (Open response on survey) I think AACs are underused since people do not have proper training. My PECS training really shaped my beliefs, and I think they should be much more widely used. (Open response on survey)
Minimal to no training during higher education	So during my education I received no training on AAC devices. I think our professor mentioned it once as like, yeah, and some kids use this to talk, but that was pretty much the extent of my experience with AACs during my master’s course work. So both during my master’s and what I’ve done of my PhD, I didn’t directly learn anything about AAC and like through coursework. (Respondent 1, 24 June 2024, 12:00 p.m.) I want to say that like one of the courses, a pretty sizable chunk was devoted to it. So I want to say like several lectures at least, maybe 3 or 4. (Respondent 5, 1 July 2024, 10:00 a.m.)
Trainings are hard to find	You gotta really comb through it. You have to go back to like when you’re first in college, and they’re teaching you about deep searching on Google and it’s like, well, that was a really long time ago. (Respondent 4, 27 June 2024, 6 p.m.) There’s not a lot of BACB like CEUs that I found that are related to AAC devices. So even the CEUs I took were actually for SLPs. (Respondent 2, 24 June 2024, 2:30 p.m.) There’s just not a lot of, I think, instruction or continuing education focus strictly on AAC device usage correctly from like BCBAs. (Respondent 2, 24 June 2024, 2:30 p.m.)
Available trainings are insufficient	And specifically, that LAMP training as well like it was, wasn’t it wasn’t meant to be used by me, right? Because it was like for speech, language pathologists. But I learned a lot just about how like their philosophy and how they implement it and can kind of apply it in more of like a behavioral kind of perspective. (Respondent 3, 25 June 2024, 12 p.m.) Cause a lot of the AAC CEUs that I’ve seen and have done, I’ve only done one or two, but they weren’t detailed enough or specific enough for me to apply those skills in my setting versus the one for SLPs. (Respondent 2, 24 June 2024, 2:30 p.m.)
Want more training	So I just took it upon myself to do some of that. Like CEUs. (Respondent 2, 24 June 2024, 2:30 p.m.) We have more and more kids coming in with an AAC device like that’s been taught since they were like 18 months through their regional center program. And so like I would be the barrier to their trajectory of growth if I don’t know how to utilize it. (Respondent 2, 24 June 2024, 2:30 p.m.)
**Facilitators**	Defined as the things that help increase AAC knowledge or comfortability using AAC, including collaboration and hands on learning. Collaboration refers to working with other professionals to provide desired outcomes for a client and hands on learning includes learning through experience and receiving feedback in a natural setting.
Hands on learning increases understanding	I mean, I think, really, it’s just getting connected with people that have that experience. So whether it’s an SLP or like another BCBA that’s implemented it a lot. I really think it’s like that hands on experience and access. (Respondent 5, 1 July 2024, 10:00 a.m.) I think definitely, seeing it in action has been helpful. And then having again the SLP shadow me, or someone who’s more well versed in it shadow me to see if I’m doing it correctly. (Respondent 2, 24 June 2024, 2:30 p.m.)
Collaboration with families	A lot of times it involves partnership with the parents as well and really having them on board with the use and the implementation. (Respondent 5, 1 July 2024, 10:00 a.m.) I’ve been able to work directly with speech therapists as well as families and teachers on implementation of AAC devices. And that’s where I’ve gotten most of my information from and training on it and how to implement it. And I mean, it’s really helped a lot. I’ve learned way more in the last 2 years than I did you know, during my education and fieldwork hours. (Respondent 4, 27 June 2024, 6 p.m.)
Collaboration with experts in AAC	Have one person be like an expert in it and get the training and kind of just like and give it out to the rest of the staff at the company. (Respondent 3, 25 June 2024, 12 p.m.) And then hopefully, if you have someone in your immediate community like a BCBA who is familiar with the AAC device just to like, collaborate and learn from them, too. (Respondent 2, 24 June 2024, 2:30 p.m.)
Collaboration with SLP	And an SLP to test for, that company that we used like SLP has to do like a whole assessment of which app is the best, and things like that to make sure it’s individualized to that specific client. (Respondent 3, 25 June 2024,12 p.m.) I think referencing the true experts, who are usually the SLPs, and making sure that you connect with that person. (Respondent 1, 24 June 2024, 12:00 p.m.) If I didn’t have these SLPs a phone call away where I can collaborate with them, I don’t know where I would have gotten the AAC training. (Respondent 2, 24 June 2024, 2:30 p.m.)
SLPs help access devices	She’s [the SLP] helped get families connected with like getting even like starting to get an AAC and connected with programs that can get them like an iPad or things like that. (Respondent 5, 1 July 2024,10:00 a.m.)
**Barriers**	Defined as anything that makes using or understanding AAC difficult, including a personal lack of understanding, the general public’s lack of understanding, other professionals being unwilling to collaborate, and an uncertainty about how AAC fits within a BCBA’s scope of practice or competence. Scope of practice refers to the activities that the BCBA credential allows you to do legally, while scope of competence refers to the activities an individual practitioner has experience with and training in.
Limited, client specific understanding	And so with that client, I learned that client specific use of the AAC device… I don’t know if I have the confidence to like use AAC device for any child. (Respondent 2, 24 June 2024, 2:30 p.m.)
Difficult to use	And there’s so many different vocabularies and like apps. And how do you choose? (Respondent 3, 25 June 2024,12 p.m.) I think that it’s a little clunky, and maybe not super intuitive to everyone, like to every child or adult that needs it. (Respondent 1, 24 June 2024, 12:00 p.m.)
Requires time and self-motivation to learn	I think it’s important to like actively seek out that education on it as well. So I’ve tried to take some additional like CEUs on that as well. (Respondent 4, 27 June 2024, 6 p.m.) So to be able to use that fluidly in our session is something I’m taking upon myself to learn now, just for this client. (Respondent 2, 24 June 2024, 2:30 p.m.)
No obvious best practice	I don’t see one like best practice from any of those fields. I mean our field, either. (Respondent 3, 25 June 2024,12 p.m.)
People don’t understand AAC	And then partnering with the family of like, it’s got to come every day, and we’ve got to send it back every day. (Respondent 5, 1 July 2024,10:00 a.m.) Going off that as well, so yeah, I think, like, socially acceptable—that seems to be a bit of a barrier. Also, I think probably other people’s understanding of what and how to use them. (Respondent 1, 24 June 2024, 12:00 p.m.)
Aspects of AAC perceived as not within their scope of competence	I feel like it’s kind of out of my scope at the moment to really suggest [starting an AAC]. (Respondent 4, 27 June 2024, 6 p.m.) But yeah, we can get into a situation where we feel like we’re all things speech and OT and BCBA and advocate. And we’re really not. (Respondent 1, 24 June 2024, 12:00 p.m.) So I think that’s the one thing I would recommend just open mind and opportunities to learn something different that are maybe not in our scope of practice. (Respondent 2, 24 June 2024, 2:30 p.m.)
ABA has a stigma	But here in [city], because parents love to go on the rabbit hole, ABA does not have a good rep, and it’s misconstrued a lot like they don’t understand like, yes, ABA’s history is not great, but the science of it is not what they’re talking about. It’s really just the culture of it. And so there’s a lot of hesitation to work with ABA fields and BCBAs in general… when I did the SLP CEU as a BCBA. There was a lot of, you know, negativity around that as well. So I think there wasn’t as much collaboration as I’d hoped for, because there was always there’s already this pretense of like, nope, like you’re doing the wrong thing like we’re here to do the right thing. (Respondent 2, 24 June 2024, 2:30 p.m.) But I do coordinate with like outside SLPs and outside OTs. For my clients here because we work in a group setting and when they’re not like in house SLPs and OTs, there is a bit of an hesitation already when I call them to get those collaboration and coordination of care, I think once they realize, like we are more of a preschool, and there’s a little bit more like early childhood education as our background before we even implement the science of ABA. Then—I can physically hear their voice relaxing and willing to wanting to like collaborate with me. (Respondent 2, 24 June 2024, 2:30 p.m.)

Note. AAC refers to augmentative and alternative communication. BCBA refers to Board-Certified Behavior Analyst. CEU refers to continuing education units. LAMP refers to the Language Acquisition through Motor Planning method. OT refers to occupational therapist. PECS refers to the Picture Exchange Communication System. SLP refers to speech–language pathologist.

## Data Availability

The data presented in this study are available on request from the corresponding author due to ethical restrictions.

## Supplementary Materials

The following supporting information can be downloaded at https://www.mdpi.com/article/10.3390/bs16081402/s1. Supplementary S1: Survey Questions; Supplementary S2: Interview Questions; Supplementary S3: Informed Consent Documents.

## Abbreviations

The following abbreviations are used in this manuscript:

AAC	Augmentative and Alternative Communication
ABA	Applied Behavior Analysis
BACB	Behavior Analyst Certification Board
BCaBA	Board-Certified Assistant Behavior Analyst
BCBA	Board-Certified Behavior Analyst
CEU	Continuing Education Unit
SLP	Speech–Language Pathologist
QBA	Qualified Behavior Analyst

## References

[B1-behavsci-16-01402] Ahsan K., Akbar S., Kam B., Abdulrahman M. D.-A. (2023). Implementation of micro-credentials in higher education: A systematic literature review. Education and Information Technologies.

[B2-behavsci-16-01402] Andzik N. R., Schaefer J. M., Christensen V. L. (2021). The effects of teacher-delivered behavior skills training on paraeducators’ use of a communication intervention for a student with autism who uses AAC. Augmentative and Alternative Communication.

[B3-behavsci-16-01402] Barman B. E., Dubasik V. L., Brackenbury T., Colcord D. J. (2023). Graduate students’ perceived preparedness and confidence to work with individuals who use augmentative and alternative communication. American Journal of Speech-Language Pathology.

[B4-behavsci-16-01402] Beaulieu L., Addington J., Almeida D. (2019). Behavior analysts’ training and practices regarding cultural diversity: The case for culturally competent care. Behavior Analysis in Practice.

[B5-behavsci-16-01402] Behavior Analyst Certification Board (2020). Ethics code for behavior analysts.

[B6-behavsci-16-01402] Behavior Analyst Certification Board (2024). BCBA test content outline *(6th ed.)*.

[B7-behavsci-16-01402] Behavior Analyst Certification Board (2025). BACB certificant data.

[B8-behavsci-16-01402] Beukelman D. R., Light J. C. (2020). Augmentative & alternative communication: Supporting children and adults with complex communication needs.

[B9-behavsci-16-01402] Bondy A. S., Frost L. A. (1994). The picture exchange communication system. Focus on Autistic Behavior.

[B10-behavsci-16-01402] Braun V., Clarke V. (2006). Using thematic analysis in psychology. Qualitative Research in Psychology.

[B11-behavsci-16-01402] Chung Y.-C., Stoner J. B. (2016). A meta-synthesis of team members’ voices: What we need and what we do to support students who use AAC. Augmentative and Alternative Communication.

[B12-behavsci-16-01402] Clarke K. A., Williams D. L. (2020). Instruction using augmentative and alternative communication supports: Description of current practices by speech-language pathologists who work with children with autism spectrum disorder. American Journal of Speech-Language Pathology.

[B13-behavsci-16-01402] Cooper J. O., Heron T. E., Heward W. L. (2020). Applied behavior analysis.

[B14-behavsci-16-01402] Creswell J. W., Plano Clark V. L. (2018). Designing and conducting mixed methods research.

[B15-behavsci-16-01402] Da Fonte M. A., Boesch M. C., DeLuca E. R., Papp S. K., Mohler A. E., Holmes E. E., Clouse K. A., Young R. D., Urbano R. (2022). Current preparation status in AAC: Perspectives of special education teachers in the United States. Augmentative and Alternative Communication.

[B16-behavsci-16-01402] Douglas S. N., Bowles R., Plavnick J., Sun T., Dunkel-Jackson S. M., Bagawan A. (2024). Iterative pilot testing of the POWR intervention: Online training to support paraeducator implementation of augmentative and alternative communication strategies. Journal of Special Education Technology.

[B17-behavsci-16-01402] Fisher W. W., Luczynski K. C., Blowers A. P., Vosters M. E., Pisman M. D., Craig A. R., Hood S. A., Machado M. A., Lesser A. D., Piazza C. C. (2020). A randomized clinical trial of a virtual-training program for teaching applied-behavior-analysis skills to parents of children with autism spectrum disorder. Journal of Applied Behavior Analysis.

[B18-behavsci-16-01402] Genc-Tosun D., Kurt O., Pektas-Karabekir E. (2025). Using an adapted picture exchange communication systems protocol for teaching children with autism spectrum disorder to make requests via a speech generating device: Preliminary findings. International Journal of Developmental Disabilities.

[B19-behavsci-16-01402] Gilroy S. P., McCleery J. P., Leader G. (2017). Systematic review of methods for teaching social and communicative behavior with high-tech augmentative and alternative communication modalities. Review Journal of Autism and Developmental Disorders.

[B20-behavsci-16-01402] Gitimoghaddam M., Chichkine N., McArthur L., Sangha S. S., Symington V. (2022). Applied behavior analysis in children and youth with autism spectrum disorders: A scoping review. Perspectives on Behavior Science.

[B21-behavsci-16-01402] Koenig M., Gerenser J. (2006). SLP-ABA: Collaborating to support individuals with communication impairments. The Journal of Speech and Language Pathology—Applied Behavior Analysis.

[B22-behavsci-16-01402] Kranak M. P., Andzik N. R., Falligant J. M. (2023). Evaluating sources of continuing education and professional development used by behavior analysts. Behavior Analysis in Practice.

[B23-behavsci-16-01402] LaFrance D. L., Weiss M. J., Kazemi E., Gerenser J., Dobres J. (2019). Multidisciplinary teaming: Enhancing collaboration through increased understanding. Behavior Analysis in Practice.

[B24-behavsci-16-01402] Lane J. D., Brown J. A. (2023). Child communication research and practice: Collaborative roles for behavior analysts and speech-language pathologists. Policy Insights from the Behavioral and Brain Sciences.

[B25-behavsci-16-01402] Lorah E. R., Holyfield C., Griffen B., Caldwell N. (2024). A systematic review of evidence-based instruction for individuals with autism using mobile augmentative and alternative communication technology. Review Journal of Autism and Developmental Disorders.

[B26-behavsci-16-01402] Maffei-Almodovar L., Sturmey P., Jessel J. (2025). Effectiveness of pyramidal training on staff acquisition of five behavior analytic procedures in the school. Journal of Behavioral Education.

[B27-behavsci-16-01402] Marshall K. B., Bowman K. S., Tereshko L., Suarez V. D., Schreck K. A., Zane T., Leaf J. B. (2023). Behavior analysts’ use of treatments for individuals with autism: Trends within the field. Behavior Analysis in Practice.

[B28-behavsci-16-01402] McConomy M. A., Root J., Wade T. (2022). Using task analysis to support inclusion and assessment in the classroom. Teaching Exceptional Children.

[B29-behavsci-16-01402] McCoy A., McNaughton D. (2021). Effects of online training on educators’ knowledge and use of system of least prompts to support augmentative and alternative communication. Journal of Behavioral Education.

[B30-behavsci-16-01402] McGill N., McLeod S., Crowe K., Wang C., Hopf S. C. (2021). Waiting lists and prioritization of children for services: Speech-language pathologists’ perspectives. Journal of Communication Disorders.

[B31-behavsci-16-01402] Mirenda P., Iacono T. (2009). Autism spectrum disorders and AAC.

[B32-behavsci-16-01402] Palinkas L. A., Horwitz S. M., Green C. A., Wisdom J. P., Duan N., Hoagwood K. (2015). Purposeful sampling for qualitative data collection and analysis in mixed method implementation research. Administration and Policy in Mental Health and Mental Health Services Research.

[B33-behavsci-16-01402] Pruneti C., Coscioni G., Guidotti S. (2024). Evaluation of the effectiveness of behavioral interventions for autism spectrum disorders: A systematic review of randomized controlled trials and quasi-experimental studies. Clinical Child Psychology and Psychiatry.

[B34-behavsci-16-01402] Pyramid Educational Consultants (n.d.). Trainings and talks.

[B35-behavsci-16-01402] Qualtrics (2024). Qualtrics *(Version 7.2024)*.

[B36-behavsci-16-01402] Rodgers M., Simmonds M., Marshall D., Hodgson R., Stewart L. A., Rai D., Wright K., Ben-Itzchak E., Eikeseth S., Eldevik S., Kovshoff H., Magiati I., Osborne L. A., Reed P., Vivanti G., Zachor D., Couteur A. L. (2021). Intensive behavioural interventions based on applied behaviour analysis for young children with autism: An international collaborative individual participant data meta-analysis. Autism.

[B37-behavsci-16-01402] Rose V., Trembath D., Keen D., Paynter J. (2016). The proportion of minimally verbal children with autism spectrum disorder in a community-based early intervention programme. Journal of Intellectual Disability Research.

[B38-behavsci-16-01402] Slocum T. A., Detrich R., Wilczynski S. M., Spencer T. D., Lewis T., Wolfe K. (2014). The evidence-based practice of applied behavior analysis. The Behavior Analyst.

[B39-behavsci-16-01402] Speech Pathology Applied Behavior Analysis Special Interest Group (2025). Speech pathology applied behavior analysis (SPABA) special interest group (SIG) of ABAI business meeting *[Powerpoint slides]*.

[B40-behavsci-16-01402] Spencer T. D., Slim L., Cardon T., Morgan L. (2020). Interprofessional collaborative practice between behavior analysts and speech-language pathologists.

[B41-behavsci-16-01402] Tager-Flusberg H., Kasari C. (2013). Minimally verbal school-aged children with autism spectrum disorder: The neglected end of the spectrum. Autism Research.

[B42-behavsci-16-01402] Uthoff S. A. K., Zinkevich A., Boenisch J., Sachse S. K., Bernasconi T., Ansmann L. (2021). Collaboration between stakeholders involved in augmentative and alternative communication (AAC) care of people without natural speech. Journal of Interprofessional Care.

[B43-behavsci-16-01402] Wormnæs S., Abdel Malek Y. (2004). Egyptian speech therapists want more knowledge about augmentative and alternative communication. Augmentative and Alternative Communication.

