# Supplementary material for: Exploring Knowledge and Sources of Information on Augmentative and Alternative Communication Among Board-Certified Behavior Analysts
Source: Behav Sci (Basel). 2026 Aug 15;16(8):1402. doi: 10.3390/bs16081402 (PMC13510283; doi:10.3390/bs16081402)
Supplement: Supplementary file 1 [file behavsci-16-01402-s001.zip › behavsci-4401195-supplementary.pdf]

Supplementary S1

Survey Template

Informed Consent Here

<u>Inclusion Criteria</u>

1. Are you a Board Certified Behavior Analyst (BCBA) or Board Certified Behavior Analyst-Doctoral (BCBA-D)? Yes No
2. Are you at least 18 years old? Yes No

<u>Demographics</u>

3. Select your age.
   - Under 30 years old
   - 30–39
   - 40–49
   - 50–59
   - 60–69
   - 70–79
   - Greater than 80

4. Select your gender
   - Female
   - Male
   - Nonbinary/third gender
   - Prefer to self-describe: _________
   - Prefer not to say
5. Select your race (select all that apply).
   - White
   - Black/African American
   - Asian
   - Native
   - Hawaiian/other Pacific Islander
   - American Indian/Alaskan Native
   - Middle Eastern or North African
   - Prefer to self-describe: __________

6. Select your ethnicity:
   - Hispanic, Latino, or Spanish origin
   - Non-Hispanic, Latino, or Spanish origin

7. What is your primary role as a BCBA, or BCBA-D?
   - Practitioner (direct services)
   - Supervisor
   - Administrator
   - Lecturer/instructor
   - Professor/researcher

Other:

8. How many years have you been a BCBA?

<1
1-5
5-10
10-15
15-20
20+

9. What population do you primarily work with?

Autism spectrum disorder
Intellectual disability
Special education
Emotional or behavioral disorders
Mental health
General education
Brain injury
Typically developing
Gerontology
Employees
Child welfare
Other:

10. What is your primary work setting?

Public school
Private school
Client’s home
Center or clinic
College or university
Residential facility
Hospital Community
Other: _____________

11. Which field is your master’s degree in?

Applied behavior analysis
Behavior analysis
Psychology
Education
Other:

12. Which field is your PhD in?

Applied behavior analysis
Behavior analysis
Psychology
Education
Other:
Not Applicable. I do not have PhD

Augmentative and Alternative Communication (AAC) can be used to help individuals communicate without using vocal speech. AAC includes a variety of techniques and tools that can help an individual communicate. For the purpose of this study, we will be focusing on aided uses of AAC, or AAC that requires some form of external tool. For example, Picture Communication Exchange System (PECS) or communications apps such as Proloquo2go are commonly used forms of AAC in the field of ABA.

13. How many total years of experience do you have working providing AAC to at least one individual (includes work as an RBT, teacher, or other relevant professional experience)?
    - <1
    - 1-5
    - 5-10
    - 10-15
    - 15-20
    - 20+

14. How *comfortable* do you feel teaching someone how to use augmentative and alternative communication strategies (AAC)?
    - Very uncomfortable
    - Uncomfortable
    - Fairly comfortable
    - Moderately comfortable
    - Very comfortable

    Please elaborate on your response above:

15. How *comfortable* do you feel collaborating with someone to determine how to use augmentative and alternative communication strategies (AAC)?
    - Very uncomfortable
    - Uncomfortable
    - Fairly comfortable
    - Moderately comfortable
    - Very comfortable

    Please elaborate on your response above:

16. How *easy or difficult* do you feel it is to find someone to collaborate with on the use of augmentative and alternative communication strategies (AAC)?
    - Very difficult
    - Difficult
    - Somewhat easy
    - Moderately easy
    - Very easy

    Please elaborate on your response above:

17. How *skilled* do you feel teaching someone how to use AAC?

    Very unskilled
    Unskilled
    Fairly Skilled
    Moderately Skilled
    Very Skilled

    Please elaborate on your response above:

18. How *important* do you feel it is to receive training and education on how to use AAC?

    Not important
    Somewhat important
    Neutral
    Moderately important
    Very important

    Please elaborate on your response above:

19. To what extent do you have knowledge and skills in AAC assessment?

    None
    Little (I have heard of it, but have not implemented it)
    Some (I have some knowledge and implemented it a few times)
    Moderate (I understand it and implement regularly)
    Extensive (I am proficient in knowledge and skills and could teach someone else)

20. To what extent do you have knowledge and skills in AAC instruction?

    None
    Little (I have heard of it, but have not implemented it)
    Some (I have some knowledge and implemented it a few times)
    Moderate (I understand it and implement regularly)
    Extensive (I am proficient in knowledge and skills and could teach someone else)

21. To what extent do you have knowledge and skills in vocabulary selection?

    None
    Little (I have heard of it, but have not implemented it)
    Some (I have some knowledge and implemented it a few times)
    Moderate (I understand it and implement regularly)
    Extensive (I am proficient in knowledge and skills and could teach someone else)

22. To what extent do you have knowledge and skills in types of symbols?

    None
    Little (I have heard of it, but have not implemented it)
    Some (I have some knowledge and implemented it a few times)
    Moderate (I understand it and implement regularly)
    Extensive (I am proficient in knowledge and skills and could teach someone else)

23. To what extent do you have knowledge and skills in no/low tech AAC systems (e.g. PECS)?

    None

Little (I have heard of it, but have not implemented it)
Some (I have some knowledge and implemented it a few times)
Moderate (I understand it and implement regularly)
Extensive (I am proficient in knowledge and skills and could teach someone else)

24. To what extent do you have knowledge and skills in high tech AAC systems (e.g. speech generative devices, apps such as Proloquo2Go or TD Snap)?
    None
    Little (I have heard of it, but have not implemented it)
    Some (I have some knowledge and implemented it a few times)
    Moderate (I understand it and implement regularly)
    Extensive (I am proficient in knowledge and skills and could teach someone else)

Please elaborate on your responses above (questions 19-24):

25. During your *master's degree education*, how much material in your *behavior-analytic coursework* was dedicated to teaching you to use AAC?
    None
    Little (one article)
    Some (part of a class period)
    Moderate (one class period)
    Extensive (several class periods or more)
    Not sure

26. During your *master's degree education*, how much material in your *non-behavior-analytic coursework* was dedicated to teaching you how to use AAC?
    None
    Little (one article)
    Some (part of a class period)
    Moderate (one class period)
    Extensive (several class periods or more)
    Not sure

27. During your *doctoral degree education*, how much material in your *behavior-analytic coursework* was dedicated to teaching you how to use AAC?
    None
    Little (one article)
    Some (part of a class period)
    Moderate (one class period)
    Extensive (several class periods or more)
    Not sure
    N/A

28. During your *doctoral degree education*, how much material in your *non-behavior-analytic coursework* was dedicated to teaching you how to use AAC?
    None
    Little (one article)

Some (part of a class period)
Moderate (one class period)
Extensive (several class periods or more)
Not sure
N/A

29. During your *master's degree* education, how much *hands-on training* (e.g., fieldwork, practicum) was dedicated to teaching you how to use AAC?
    None
    Little (one instance)
    Some (one training)
    Moderate (several trainings)
    Extensive (several trainings and trained others)

30. During your *doctoral degree* education, how much *hands-on training* (e.g., fieldwork, practicum) was dedicated to teaching you how to use AAC?
    None
    Little (one instance)
    Some (one training)
    Moderate (several trainings)
    Extensive (several trainings and trained others)
    N/A

31. Since becoming certified, how much continuing education (CE) have you received teaching you how to use AAC?
    None
    Little (1-3 credits)
    Some (3-5 credits)
    Moderate (5-10 credits)
    Extensive (11+ credits)

32. Since becoming certified, how many continuing education (CE) opportunities have you seen offered at behavior-analytic conferences or online teaching you how to use AAC?
    None
    1-2 per year
    3-4 per year
    5-8 per year
    8+ per year

33. How much training does your employer provide you on how to use AAC?
    None
    Little (I was shown how to by a colleague a couple of times)
    Some (I received one training session)
    Moderate (I received several training sessions)
    Extensive (I received several training sessions and extensive feedback while implementing AAC)

34. To what extent did you obtain your knowledge and skills through personal experience?

None
Little (I worked with one individual who use AAC)
Some (I worked with a few individuals who use AAC)
Moderate (I have frequently worked with individuals who use AAC)
Extensive (I have worked with individuals with AAC throughout my whole career)

35. To what extent did you obtain your knowledge and skills through collaboration with other professionals, such as speech language pathologists (SLPs) or teachers?
    None
    Little (I had one interaction with another professional regarding AAC)
    Some (I received a full training from another professional regarding AAC)
    Moderate (I have collaborated with other professionals several times regarding AAC)
    Extensive (I frequently collaborate with other professionals regarding AAC)

36. To what extent did you obtain your knowledge and skills through other sources (colleagues, TV presentations, journals, books)?
    None
    Little (I read one article on AAC)
    Some (I have read a couple articles or a book on AAC)
    Moderate (I have read many articles or a couple books on AAC)
    Extensive (I have read many articles and read several books on AAC)

    Please elaborate on your response above:

38. What do you wish you knew or had competence in regarding AAC? (Open response question)

39. Any other opinions about and/or experiences with AAC? (Open response question)

If you would like to be contacted for a virtual interview, please provide your email address:

Please enter your email address to receive an electronic gift card:

Supplementary S2

Interview questions

1. Tell me about your experience with AAC that you received during your education.
2. Tell me about your experience with AAC that you received through professional development.
3. Tell me about your experience with implementing AAC.
    a. (Potential follow up question) Tell me about your experience with collaborating with other professionals about AAC.
4. Tell me about the barriers to implementing AAC
5. Tell me about the facilitators (or aspects that help) to implementing AAC.
6. What do you recommend for other BCBAs that want to learn more about AAC?

Supplementary S3

Informed Consent Documents

Protocol #14326
IRB Exemption Date: May 17, 2024
Consent Document Expires: August 30, 2024

Research UtahStateUniversity

v.2.5

Letter of Information

Augmentative and Alternative Communication in Behavior Analysis: A Survey of Sources and Understanding

You are invited to participate in a research study by Dr. Sophia D'Agostino, an Assistant Professor of Early Childhood Special Education in the Department of Special Education and Rehabilitation Counseling at Utah State University. The purpose of this research is to investigate the training and understanding that behavior analysts have regarding the use of Augmentative and Alternative Communication (AAC). Specifically, we are interested in learning about where behavior analysts receive their training on AAC, what levels of understanding they may have regarding concepts related to using and teaching AAC, and their comfort level teaching others to use AAC. You are being asked to participate in this research because you are a BCBA or BCBA-D, are at least 18 years old, and live within the United States. If you take part in this study, you will be asked to complete a 30-minute electronic survey. At the end of this study if you meet the minimum participation requirements, you may be invited to participate in an interview for the second phase of this study if you choose.

Your participation in this study is voluntary and you may withdraw your participation at any time by ending the survey for any reason.

The possible risks of participating in this study include loss of confidentiality. This study has been designed to learn more about behavior analysts' understanding and training on the use of AAC. This will create a better understanding of potential gaps in available training on the use of AAC so training opportunities can be created.

We will make every effort to ensure that the information you provide remains confidential. We will not reveal your identity in any publications, presentations, or reports resulting from this research study.

We will collect your information through Qualtrics, an online survey system. Online activities always carry a risk of a data breach, but we will use systems and processes that minimize breach opportunities. This survey data will be securely stored in a restricted-access folder on Box.com, an encrypted, cloud-based storage system.

The study team is using fraud protection mechanisms to ensure genuine participation in this study. If your response is detected as fraudulent from one of those features, the study team may withdraw your response, or we will need to take extra steps to confirm your identity and responses.

For your participation in this research study, you will receive a $10 amazon.com e-gift card for completion (at least 90%) of the online survey.

You can decline to participate in any part of this study for any reason and can end your participation at any time. If you have any questions about this study, you can contact Sophia D'Agostino, Assistant Professor; Phone: 435-797-3240; e-mail: Sophia.dagostino@usu.edu. Thank you again for your time and consideration. If you have any concerns about this study, please contact Utah State University's Human Research Protection Office at (435) 797-0567 or irb@usu.edu.

By continuing to the next page of the survey you agree that you are 18 years of age or older and wish to participate. You agree that you understand the risks and benefits of participation, and that you know what you are being asked to do. You also agree that if you have contacted the research team with any questions about your participation and are clear on how to stop your participation in this study if you choose to do so. Please be sure to retain a copy of this form for your records.

Research
UtahStateUniversity

v.2.5
Informed Consent

Augmentative and Alternative Communication in Behavior Analysis: A Survey of Sources and Understanding

You are invited to participate in a research study by Dr. Sophia D'Agostino, an Assistant Professor of Early Childhood Special Education in the Department of Special Education and Rehabilitation Counseling at Utah State University. The purpose of this research is to investigate the training and understanding that behavior analysts have regarding the use of Augmentative and Alternative Communication (AAC). Specifically, we are interested in learning about where behavior analysts receive their training on AAC, what levels of understanding they may have regarding concepts related to using and teaching AAC, and their comfort level teaching others to use AAC. If you take part in this study, you will be asked to participate in a Zoom interview (approximately 45 minutes).

Your participation in this study is voluntary and you may withdraw your participation at any time by ending the Zoom call, emailing with a request to be removed, or through the absence of a reply greater than 3 weeks.

The possible risks of participating in this study include loss of confidentiality. This study has been designed to learn more about behavior analysts' understanding and training on the use of AAC. This will create a better understanding of potential gaps in available training on the use of AAC so training opportunities can be created.

We will make every effort to ensure that the information you provide remains confidential. We will not reveal your identity in any publications, presentations, or reports resulting from this research study. However, it may be possible for someone to recognize the specifics you share with us.

We will collect your information through Zoom, a videoconferencing system. Online activities always carry a risk of a data breach, but we will use systems and processes that minimize breach opportunities. This interview data will be securely stored in a restricted-access folder on Box.com, an encrypted, cloud-based storage system. Once you've approved the transcript, we will delete the recording.

There will be no additional compensation for participating in this interview.

You can decline to participate in any part of this study for any reason and can end your participation at any time. If you have any questions about this study, you can contact Sophia D'Agostino, Assistant Professor; Phone: 435-797-3240; e-mail: Sophia.dagostino@usu.edu. Thank you again for your time and consideration. If you have any concerns about this study, please contact Utah State University's Human Research Protection Office at (435) 797-0567 or irb@usu.edu.

By signing below, you agree that you are 18 years of age or older and wish to participate. You agree that you understand the risks and benefits of participation, and that you know what you are being asked to do. You also agree that if you have contacted the research team with any questions about your participation and are clear on how to stop your participation in this study if you choose to do so. Please be sure to retain a copy of this form for your records.

____________________	____________________	__________
Participant's Signature	Participant's Name, Printed	Date
